# A method to predict breast cancer stage using Medicare claims

**DOI:** 10.1186/1742-5573-7-1

**Published:** 2010-01-15

**Authors:** Grace L Smith, Ya-Chen T Shih, Sharon H Giordano, Benjamin D Smith, Thomas A Buchholz

**Affiliations:** 1 Department of Radiation Oncology, The University of Texas MD Anderson Cancer Center, 1515 Holcombe Blvd Houston, Texas 77030, USA; 2 Department of Biostatistics, Section of Health Services Research, The University of Texas MD Anderson Cancer Center, 1515 Holcombe Blvd, Houston, Texas 77030, USA; 3 Department of Breast Medical Oncology, The University of Texas MD Anderson Cancer Center, 1515 Holcombe Blvd, Houston, Texas 77030, USA; 4 Radiation Oncology Flight, Wilford Hall Medical Center, Lackland Air Force Base, San Antonio, Texas 78236, USA

## Abstract

**Background:**

In epidemiologic studies, cancer stage is an important predictor of outcomes. However, cancer stage is typically unavailable in medical insurance claims datasets, thus limiting the usefulness of such data for epidemiologic studies. Therefore, we sought to develop an algorithm to predict cancer stage based on covariates available from claims-based data.

**Methods:**

We identified a cohort of 77,306 women age ≥ 66 years with stage I-IV breast cancer, using the Surveillence Epidemiology and End Results (SEER)-Medicare database. We formulated an algorithm to predict cancer stage using covariates (demographic, tumor, and treatment characteristics) obtained from claims. Logistic regression models derived prediction equations in a training set, and equations' test characteristics (sensitivity, specificity, positive predictive value (PPV), and negative predictive value [NPV]) were calculated in a validation set.

**Results:**

Of the entire sample of women diagnosed with invasive breast cancer, 51% had stage I; 26% stage II; 11% stage III; and 4% stage IV disease. The equation predicting stage IV disease achieved sensitivity of 81%, specificity 89%, positive predictive value (PPV) 24%, and negative predictive value (NPV) 99%, while the equation distinguishing stage I/II from stage III disease achieved sensitivity 83%, specificity 78%, PPV 98%, and NPV 31%. Combined, the equations most accurately identified early stage disease and ascertained a sample in which 98% of patients were stage I or II.

**Conclusions:**

A claims-based algorithm was utilized to predict breast cancer stage, and was particularly successful when used to identify early stage disease. These prediction equations may be applied in future studies of breast cancer patients, substantially improving the utility of claims-based studies in this group. This method may similarly be employed to develop algorithms permitting claims-based epidemiologic studies of patients with other cancers.

## Background

Administrative medical insurance claims are an important source of population-based data used in epidemiologic studies of various diseases. Specifically, in older patients, national Medicare data have been useful for the study of many conditions, including myocardial infarction, heart failure, chronic kidney disease, Parkinson's disease, and venous thromboembolism [1-5]. However, for studying cancer, the use of national Medicare data has, to date, been limited. Medicare claims data are clearly recognized as potentially a rich source for cancer epidemiology and outcomes research, and in fact demonstrate acceptable validity for identifying cancer diagnoses and treatment patterns [6-13]. Unfortunately, the lack of cancer stage data in Medicare claims remains a major limiting factor in maximizing the utility of these datasets for retrospective, outcomes-based research in cancer patients[14,15]. In particular, cancer stage is a crucial predictor of disease outcome and a key factor in determining the appropriateness of treatment. For example, in breast cancer patients, stage is associated with overall and disease-free survival and, furthermore, stage influences treatment decisions such as selection and timing of surgery, radiotherapy, and chemotherapy[16]. Epidemiologic studies of cancer patients typically employ stage variables as covariates or as inclusion and exclusion criteria, and thus it is essential to develop accurate algorithms to account for cancer stage in studies using claims data.

Surprisingly, the need to derive such algorithms has largely been ignored in the literature. Only one prior study has developed a claims-based algorithm to predict stage in breast cancer patients. Cooper et al. used the Surveillance Epidemiology and End Results (SEER)-Medicare database, and authors reported that their claims-based, single-predictor models were insufficient for identifying patients with local, regional, and distant stage disease. Sensitivity of these models for distinguishing local from regional and distant disease was low--for example, in breast cancer patient samples, only approximately 60%[17]. In the decade since the prior algorithm was derived, no other algorithm has been presented in the literature attempting to improve cancer stage classification using claims data. Thus studies have continued to apply the algorithm by Cooper and colleagues to derive cancer stage variables, despite the recognized limitations of this algorithm [18,19] and the introduction of measurement errors into such analyses.

Accordingly, we sought to derive an expanded predictive algorithm based on multivariate modeling and to improve the sensitivity and specificity for identifying cancer stage, using our study sample of breast cancer patients as an illustrative case. Using available Medicare claims for breast cancer patients found in the SEER-Medicare database, we developed a prediction algorithm to identify patients with distant (stage IV) disease at diagnosis and, among patients without distant disease, a prediction algorithm to classify the extent of locoregional (stages I-III) disease.

## Methods

### Algorithm

#### Study sample

The SEER-Medicare database is comprised of a population-based cohort of Medicare beneficiaries with incident cancer identified through SEER registries, which account for up to 26% of the United States' population [20,21]. Our initial study population consisted of 150,764 women (age ≥ 65 years) diagnosed with breast cancer between 1992 and 2002 identified through SEER-Medicare. From this population, we excluded 5,217 patients with unknown SEER historic stage (as this variable indicated the presence or absence of metastases), and 19,816 with *in situ *disease (as we intended to focus only on invasive disease). We further excluded 47,114 patients who did not have continuous Medicare Fee-for-Service coverage or had any HMO coverage from 12 months prior to 9 months after their diagnosis date (as claims information might be incomplete during these periods), and the 308 patients age <66, since these patients potentially would not have had comprehensive claims information to define the independent predictor covariates. We finally excluded 1,003 patients who died or were lost to follow-up within 9 months of their diagnosis date. This yielded a final sample size of 77,306 patients in our study.

#### Dependent variable: Cancer stage

The "gold standard" for identifying cancer stage at diagnosis was determined using a combination of tumor variables available through SEER. Distant disease was determined using the American Joint Committee on Cancer (AJCC) [22] historic stage as reported to SEER, which indicates tumor present in any distant site at cancer diagnosis (compared with tumor limited only to local or regional sites at diagnosis). For our analysis, patients with any distant disease were considered stage IV.

Patients without distant (stage IV) disease had local or regional AJCC historic stage. T and N classification in these patients were assigned based on SEER variables for tumor size and extent of disease. Tumor size and extent were categorized as ≤ 2 cm (T1); >2 to 5 cm (T2); >5 cm (T3); or invading into the chest wall, ribs, intercostals or serratus anterior muscles, extensive invasion into the skin, inflammatory carcinoma, or further contiguous extension into the skin (T4). Nodal disease was categorized as 0 positive lymph nodes (N0); 1-3 positive lymph nodes (N1); 4-9 positive lymph nodes (N2); or 10 or more positive lymph nodes (N3)[21]. Due to the extent of missing data in the SEER database, location of positive lymph nodes was not included in N classification. Stage I included T1N0 disease, stage II included T0N1, T1N1, T2N0, T2N1, and T3N0 disease, and stage III included T0-2N2, T3N1-2, T4N0-2, and T0-4N3 disease. These classifications are based on AJCC 2003 staging criteria[22].

#### Independent predictors

Candidate independent predictors were selected *a priori *based on statistical significance in bivariate analyses (*P *< 0.25) and clinical significance in prior studies of cancer patients[20,23-28]. Variables were defined by searching through inpatient, outpatient, and carrier Medicare claims or the denominator file for SEER-Medicare linked data for demographic variables. A comprehensive list of International Classification of Diseases, Ninth Revision (ICD-9), Common Procedural Terminology (CPT), and Revenue Center codes for each predictor are listed in Table S1, Additional file 1.

### Statistical analysis

All statistical analyses were conducted using SAS version 9.1.3 (SAS Institute Inc, Cary, NC), and all statistical tests assumed a 2-tailed α of 0.05. The University of Texas M. D. Anderson Cancer Center institutional review board deemed this study exempt from review, since the data were without identifiers.

We derived two separate logistic models and implemented the models sequentially. The first model tested the associations between predictor covariates and the dichotomous outcome of stage IV versus non-stage IV disease. Among the subset of patients who were not categorized as having stage IV disease, the second model tested the associations between predictor covariates (excluding the covariate for metastatic disease at diagnosis) and the dichotomous outcome of stage I/II (early) versus stage III disease. Outcomes were dichotomized based on clinical rationale, given that treatment of metastatic disease is palliative; and that treatment of early stage disease is distinct in that breast conserving therapy is a treatment option.

We used a split sample approach to develop and validate our logistic models. Each model was derived from the "training set," selected using simple random sampling without replacement (38,653 of 77,306 patients). Parsimonious models were then selected based on statistical significance (*P *< 0.25), clinical significance of covariates in prior studies,[20,23-28] and goodness-of-fit. Prior studies were used as an initial guide for the selection of covariates to consider. The significance cutoff (P < 0.25) was used to rule in covariates to keep. Examining the goodness-of-fit of the overall model was used to rule out covariates to exclude. In combination, these three criteria were used to select the final model.

### Testing

Patients not included in the training set constituted the "validation set". In the validation set, the parameter associated with each covariate estimated from the derivation set was applied to each patient in the validation set to calculate each patient's predictive probability (calculated probability = ) of having stage IV disease in the first model and stage I/II or stage III disease in the second model. Test characteristics were calculated for probability cutpoints between 0.05 and 0.90, using two-by-two tables. The "gold standard" for stage was considered the SEER stage; the test stage was based on the calculated probability (for example, for a probability cutpoint of 0.05, patients were predicted to have stage I/II disease if their calculated probability was ≥ 0.05, and not to have stage I/II disease if their calculated probability was <0.05).

### Combining equations

The prediction equations were then applied to isolate a sample of patients with early stage disease. Specifically, the two prediction equations were applied sequentially to the validation sample in order to identify a subset of patients with stage I/II disease. The first step used a probability cutpoint of 0.05 to exclude patients with predicted stage IV disease. The second step was applied to the subset identified in the first step and used a probability cutpoint of 0.90 to include patients with predicted stage I/II disease. These cutpoints were chosen based on their test characteristics (high sensitivity, specificity, and positive predictive value [PPV] or negative predictive value [NPV]). Finally, we also compared the test characteristics derived from our multivariate prediction equations to test characteristics derived from single-predictor equations for distant and regional disease used in a prior study,[17] to determine whether multivariate equations improved test characteristics compared with the single-predictor equations.

### Implementation

#### Example in Practice: Medicare test sample

Finally, we present an example that applies the prediction equation. We used a test sample based on a claims-only dataset, the national Medicare dataset. The national Medicare dataset includes claims data for all Medicare beneficiaries in the United States. Files contain data collected by Medicare for reimbursement of health care services for each beneficiary and include institutional (inpatient and outpatient) and non-institutional (physicians or other providers) final action claims [29]. We initially included 127,607 women (age ≥ 65) with a diagnosis claim indicating invasive breast cancer in 2003 (International Classification of Diseases, Ninth Revision (ICD-9) diagnosis code of 174) who underwent a breast-cancer related procedure. We then excluded 23,715 patients who did not have at least 2 claims (on different dates) specifying a diagnosis of invasive breast cancer between January 1, 2003 and December 31, 2004 (at least 1 claim must have occurred during 2003); 16,471 patients who had a breast cancer-related diagnosis or procedure claim between January 1, 2002, and December 31, 2002; 5,719 patients who were receiving Medicare coverage because of end-stage renal disease or disability; and 6,612 patients who lacked Part A or B coverage or who had intermittent health maintenance organization coverage in the 9 months after or in the 1 year before their breast cancer diagnosis date (of these patients, 3,561 had incomplete information in the year prior to diagnosis because they were <66 years of age); for a total sample size of 56,725 patients. This method for sample selection of incident breast cancer has been validated in a prior study [30].

In this test sample, we applied our derived algorithm and calculated the frequency of patients classified as predicted stage IV and predicted stage I/II disease. Again, for this sample, the first step used a probability cutpoint of 0.05 and the second step a probability cutpoint of 0.90. As a test of our algorithm for consistency, we compared the predicted frequencies to the actual stage distribution in two populations: 1) the SEER-Medicare population (age >65 years) and 2) the National Cancer Data Base population (age ≥ 70 years) [31].

## Results

### Patient characteristics

In 77,306 women, mean age was 76 years (standard deviation 7 years), and 94% were white. Fifty-one percent were stage I (39,147), 26% stage II (19,967), 11% stage III (8,174), 4% stage IV (3,220) and 9% with non-distant disease but T or N classification unknown (6,798). Forty-five percent were treated with breast conserving surgery, 49% with mastectomy, 44% with radiotherapy, and 18% with chemotherapy (Table 1).

**Table 1:** Study Sample Patient Characteristics, N = 77,306

Predictor Variable	% of All Patients
**Demographic**	
Age, mean (SD)	76 (7)
White race	94
**Stage**	
Stage I	51
Stage II	26
Stage III	10
Stage IV	4
Stages I-III but T or N unknown	9
**Extent of disease^a^**	
Axillary LN involvement	19
Metastatic disease	14
**Cancer treatment^a^**	
No. visits to surgeon, mean (SD)	4 (3)
No. visits to medical oncologist, mean (SD)	4 (9)
No. visits to radiation oncologist, mean (SD)	3 (5)
Imaging (CT, MRI, PET, or bone scan)	25
Radiation therapy	44
Breast conserving surgery	45
Mastectomy	49
Axillary LN dissection	72
Chemotherapy (any agent)	18
Doxorubicin	7
Paclitaxel	3
No. physician visits, mean (SD)	14 (12)
Screening mammography	78
Influenza vaccine	34
**General health status^a^**	
No. hospital admission for any cause, mean (SD)	1 (1)
Charlson comorbidity score	
0 comorbid conditions	69
1 comorbid condition	18
2 or more comorbid conditions	8
Unknown	5

Abbreviations: CT computed tomography; LN lymph nodes; MRI magnetic resonance imaging PET positron emission tomography; SD standard deviation

^a ^As indicated by Medicare claims codes; thus percentage of patients with code for metastatic disease not equal to patients with Stage IV disease.

### Prediction algorithm equations and test characteristics for probability cutpoints

Candidate covariates tested in prediction equations are listed in Table S1, Additional file 1. Parameter estimates for the covariates included in each final prediction equation are listed in Table S2, Additional file 1.

#### Stage IV

**Figure 1:**
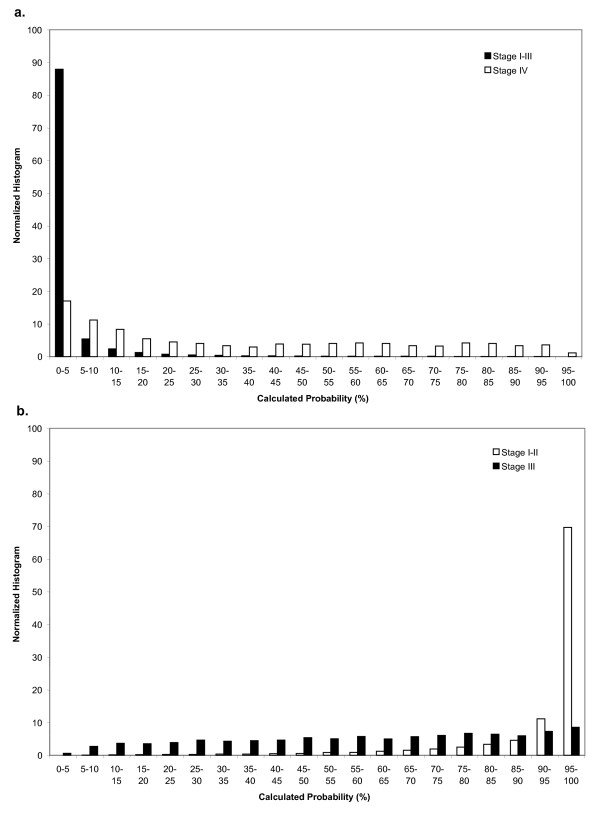
**Distribution of calculated probabilities (%) for patients with predicted stages I-III and stage IV disease (a)**. Distribution of calculated probabilities for patients with predicted stages I-II and stage III disease (b). A calculated probability of 5% corresponds to a cutpoint of 0.05. The histograms are normalized to 100%.

Fourteen percent of all patients and 73% of patients with stage IV disease had a claims code indicating possible metastatic disease. Accordingly, the single-predictor model including only this covariate had sensitivity of 73%; specificity 89%; PPV 22%; and NPV 99% for identifying stage IV disease. After including covariates (Table S2, Additional file 1) in the multivariate model, the sensitivity was 81% (95% Confidence Interval 80% - 84%) for identifying stage IV disease at a probability cutpoint of 0.05. At this cutpoint, specificity was 89% (86% - 89%); PPV 24%; (22% - 25%) and NPV 99% (99% - 99%), yielding a c-statistic of 0.93. (Table S3, Additional file 1) The distribution of calculated predicted probabilities in the validation set for patients with stage IV versus stage I-III disease is presented in Figure 1a.

#### Stages I-III

In patients with stages I-III disease, 19% had a claims code indicating axillary lymph node involvement. Specifically, 2% of patients with stage I disease, 36% with stage II disease, and 64% of patients with stage III disease had this claims code. The single-predictor model including only this covariate yielded sensitivity of 87% (specificity 61%; PPV 94%; NPV 39%) for identifying stage I/II disease and sensitivity of 61% (and specificity 87%; PPV 39%; NPV 94%) for identifying stage III disease. After including covariates (Table S2, Additional file 1) in the multivariate model, the sensitivity was 91% (90% - 92%) for identifying stage I/II disease at a probability cutpoint of 0.80; and 83% (83% - 85%) at a cutpoint of 0.90. At a cutpoint of 0.90, specificity was 78% (75% - 79%); PPV 98% (97% - 98%); and NPV 31% (30% - 34%). For identifying stage III disease, the sensitivity was 78% (75% - 79%) at a cutpoint of 0.10. At this cutpoint, specificity was 83% (83% - 85%); PPV 30% (30% - 34%); and NPV 98% (97% - 98%) (Table S3, Additional file 1). These models yielded a c-statistic of 0.88. The distribution of calculated predicted probabilities in the validation set for patients with stage I/II versus stage III disease is presented in Figure 1b.

#### Comparison with other single predictors

For comparison's sake, for identifying stage IV disease, the second most important predictor was axillary lymph node dissection. This predictor alone would yield the following test characteristics: sensitivity 67%; specificity 74%; PPV 10%; and NPV 98%. For identifying stage I/II disease, the second most important predictor was breast conserving surgery vs. mastectomy, yielding the following test characteristics: sensitivity 49%; specificity 82%; PPV 95%; and NPV 18%.

### Combining equations

**Figure 2:**
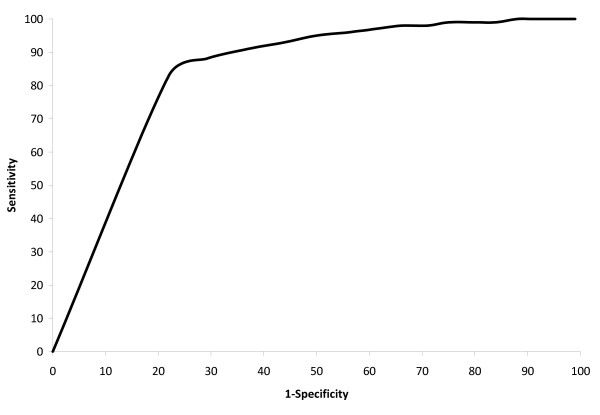
**Receiver Operating Curve (ROC) for equation to predict stage IV disease**.

**Figure 3:**
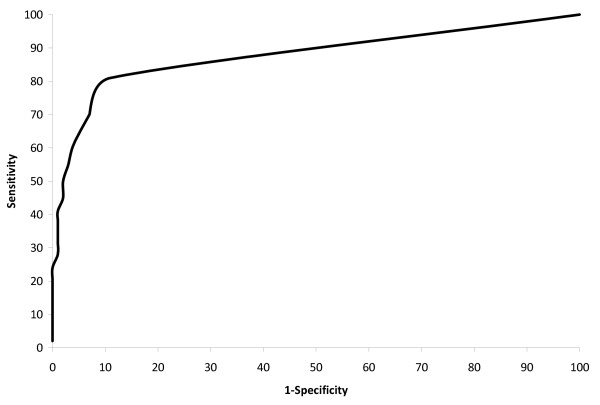
**Receiver Operating Curve (ROC) for equation to predict stage I-III disease**.

The prediction equations were most accurate for isolating patients with early stage disease. Specifically, after applying the two prediction equations sequentially to the validation sample to identify patients with predicted stage I/II disease, a subset of 23,285 patients were selected (of 38,653 patients, 36,417 were predicted to have non-stage IV disease, and of these patients, 23,285 were predicted to have stage I/II disease). The predictive sample actually consisted of 98% gold standard stage I/II disease (22,706 of 23,285), 2% stage III disease (549 of 23,285), and <1% stage IV disease (110 of 23,285). Of all patients with gold standard stage I/II disease (29,546 of 38,653 validation patients), 23% (6,840 of 29,546) were excluded (classified as other than stage I/II) as a result of the algorithm (4,604 from the first model and 2,236 from the second model). (Figure 2, Figure 3).

### Example in Practice: Medicare test sample and comparison for consistency

In our Medicare test sample, after the first predictor equation was applied, a total of 4% (2,333 of 56,725) of women were predicted as having stage IV disease. This compared favorably with the SEER-Medicare population, which included 4% (3,220 of 77,306) of women with confirmed stage IV disease; as well as the NCDB population, which included 5% (1,913 of 41,071) confirmed stage IV disease. After the second predictor equation was applied to the remainder of the test sample, a total of 79% (43,169 of 54,392) of women were predicted as having stage I/II disease. This compared favorably with the SEER-Medicare population, which included 80% (59,114 of 74,086) of women with confirmed Stage I/II disease; as well as the NCDB population, which included 84% (33,036 of 39,158) confirmed Stage I/II disease.

## Discussion

In this cohort of older breast cancer patients, Medicare claims data assisted the prediction of cancer stage. Predictor equations using claims data alone were able to achieve approximately 80% sensitivity and specificity for identifying stage IV disease as well as distinguishing stage I/II from stage III disease. Prediction models maximized NPV when distinguishing stage IV from stage I-III disease but maximized PPV when distinguishing stage I/II from III disease. With a resulting tradeoff of lower PPV in the first model and lower NPV in the second model, the algorithm was therefore found to be best suited to most accurately identify early stage disease. Specifically, an algorithm combining the two equations seeking to identify patients with early stage disease was able to achieve a sample in which 98% of patients had stage I or II disease.

Our prediction models represent an improvement over the single other previously published model. In this prior study, Cooper et al. used single-predictor equations to identify cancer stage. To identify patients with distant disease, authors tested a single variable based on claims codes for metastatic disease. This single-predictor model demonstrated 60% sensitivity and 58% PPV. To distinguish patients with local versus regional disease, authors tested a single variable based on the claim code for axillary lymph node involvement. This single-predictor model demonstrated 62% sensitivity and 85% PPV[17]. The relatively poor test characteristics from this prior study demonstrated that these single-predictor models would be insufficient for predicting stage in patients with breast cancer and suggested that claims data alone would be inadequate for epidemiologic studies of cancer patients.

In contrast, our prediction models have improved upon these test characteristics. Our single predictor model demonstrated improvement, likely in part due to a more extensive list of claims codes, with multiple covariates providing added value. Moreover, our algorithm demonstrated consistency when results were compared with population-based data from SEER-Medicare and the NCDB. There are two important future research applications of our prediction models. First, our multivariate logistic modeling method for developing a stage predictor algorithm may similarly be applied to test models and potentially develop stage prediction equations for patients diagnosed with cancers of other sites. Second, our prediction equations may also be applied directly to claims-based databases of breast cancer patients who have unknown stage. Using a combination of multiple predictors along with claims codes for metastatic disease and axillary lymph node dissection, parameter estimates and calculated probabilities can be applied to the prediction of patient breast cancer stage.

Our algorithm can therefore serve as a tool to assist in the investigation of a variety of epidemiologic research questions in breast cancer patients by allowing a sample selection of those patients with early stage disease. In addition, predicted early stage disease can be applied as a covariate. Accordingly, since disease stage may be better accounted for, claims databases of breast cancer patients may also be better applied to address such questions as the assessment of treatment utilization, geographic variation, or outcomes in patients diagnosed with early stage breast cancer. Specifically, for the stage IV prediction model, a probability cutpoint between ≥ 0.05 and ≥ 0.10 would be highly specific and sensitive for identifying patients with stage IV disease. For the stage I/II prediction model, a cutpoint between ≥ 0.80 and ≥ 0.90 would be highly specific and sensitive for distinguishing patients with stage I/II disease from patients with stage III disease.

For the identification of patients with stage IV disease, a selected probability cutpoint criterion could be translated into a dichotomous variable, and used either to select a sample of patients with stage IV disease or used in a "rule out" context, as an exclusion criterion. The high NPV in our proposed model suggests that when using these cutpoints to identify a sample limited to patients with stage I-III disease, the likelihood of misclassification bias (bias due to the inappropriate inclusion of patients with stage IV disease in the sample) would be low in a "rule-out" setting.

For distinguishing patients with stage I/II versus III disease, the probability cutpoint criterion, translated into a dichotomous variable, could be useful in various contexts, such as excluding patients with stage III disease in order to refine a study population of patients with early stage breast cancer, or creating a dichotomous covariate to adjust for potential confounding associated with stage I-III disease. The test characteristics in our analysis suggest that the combination of these prediction equations may be particularly useful in the context of identifying breast cancer patients with early stage (stage I and II) disease.

Our study has limitations to consider. First, our cohort was limited only to older patients with breast cancer. Although the variables associated with stage are likely to be similar in younger patients, exact parameter estimates may differ, and the application of these models in younger patients requires further validation. Additionally, as our predictor variables were derived from Medicare claims, these models will also require validation in other claims based data. If not all the proposed variables in our models are available, however, at a minimum, adjustment in multivariate analysis for as many possible available candidate predictors proposed in our study could be useful to improve modeling of breast cancer outcomes in future studies. Although we excluded from our parsimonious model covariates that required long-term follow-up (specifically, overall survival and mastectomy 9 or more months after diagnosis), our models still required both retrospective and prospective data for up to 1 year prior to and 1 year after the date of diagnosis. Thus, studies applying our models would be limited to patients with continuous coverage and complete claims information over this time period. The gold standard for our outcome, cancer stage, was based on pathologic variables in SEER-Medicare, though given a lack of central pathology review by the SEER program, unmeasured error may have affected the gold standard, yielding potentially less than 100% accuracy. Finally, if a sample was selected based on the algorithm, sample characteristics derived from algorithm predictor variables (for example, chemotherapy, radiotherapy, and surgery utilization) may be under- or overestimated compared with the complete patient population, depending on the direction and significance of their association with disease stage in the prediction equations.

## Conclusions

Medicare claims data can be utilized to derive a useful algorithm to predict stage in breast cancer patients. In particular, the predicted probability of early stage disease can be easily generated when applying the prediction algorithm to this patient population, thus substantially improving the utility of Medicare claims data for studying breast cancer.

## Abbreviations

AJCC: American Joint Committee on Cancer; SEER: Surveillance Epidemiology and End Results; PPV: Positive predictive value; NPV: Negative predictive value; ICD-9: International Classification of Diseases, Ninth Revision; CPT: Current Procedural Terminology.

## Competing interests

The authors declare that they have no competing interests.

## Authors' contributions

GLS helped to design the study, conduct the statistical analysis and interpret the results, and drafted the manuscript. YCTS helped to design the study and analysis, gave substantial input on the statistical design, helped to interpret the results, and gave critical revisions for manuscript content. SHG contributed to study conception, interpret the statistical analysis and results, and gave critical revisions for manuscript content. BDS helped to design the study, acquire the data, interpret the statistical analysis and results, and gave critical revisions for manuscript content. TAB contributed to study conception and design, interpret the statistical analysis and results, and gave critical revisions for manuscript content. All authors have given final approval of this manuscript.

## Supplementary Material

Additional file 1 **Table S1.** Candidate Covariates and Claims Codes. Table S2. Prediction Equations. Parameter estimates for stage IV versus stages I-III disease and for stage. I/II versus stage III disease. Table S3. Test Characteristics After Applying Prediction Equations on Validation Set Samples. Click here for file
