# Supplementary material for: A method to predict breast cancer stage using Medicare claims
Source: Epidemiol Perspect Innov. 2010 Jan 15;7:1. doi: 10.1186/1742-5573-7-1 (PMC2818641; doi:10.1186/1742-5573-7-1)
Supplement: Additional file 1 — Table S1. Candidate Covariates and Claims Codes. Table S2. Prediction Equations. Parameter estimates for stage IV versus stages I-III disease and for stage. I/II versus stage III disease. Table S3. Test Characteristics After Applying Prediction Equations on Validation Set Samples. [file 1742-5573-7-1-S1.DOC]

**Table S1. Candidate Covariates and Claims Codes**

| **Predictor Variable** | **Time period searched** | **Source File** | **ICD-9 Diagnosis** | **ICD-9 Procedure** | **CPT** | **Revenue Center** |
| --- | --- | --- | --- | --- | --- | --- |
| **Demographic** |  |  |  |  |  |  |
| Age | At diagnosis | Denominator |  |  |  |  |
| Race | At diagnosis | Denominator |  |  |  |  |
| **Extent of disease at diagnosis** |  |  |  |  |  |  |
| Axillary LN involvement | 3 mos before to 3 mos after |  | 1963 |  |  |  |
| Metastatic disease | 3 mos before to 3 mos after |  | 1962, 1965-6, 197, 1970, 1971, 1972, 1973, 1974, 1975, 1976, 1977, 1978, 198, 1980, 1981, 1982, 1983, 1984, 1985, 1986, 1987, 1988, 19881, 19882, 19889 |  |  |  |
| **Cancer treatment** |  |  |  |  |  |  |
| No. visits to surgeon | In the year after diagnosis | Carrier claims |  |  |  |  |
| No. visits to medical oncologist | In the year after diagnosis | Carrier claims |  |  |  |  |
| No. visits to radiation oncologist | In the year after diagnosis | Carrier claims |  |  |  |  |
| Imaging (CT, MRI, PET, or bone scan) | In the 3 months after diagnosis |  |  | 8801, 8703, 8741, 8891, 8896, 8874, 9218, 9214 | 70450, 70460, 70470, 70551-3, 71250, 71260, 71270, 72192-4, 74150, 74160, 74170, 76700, 78315, 78320, 78812-6, G0213-5 |  |
| Radiation therapy | In the 9 months after diagnosis |  | V580, V661, V671 | 9221-7, 9229 | 77401-77525, 77761-77799 | 0330, 0333 |
| Breast conserving surgery | In the 9 months after diagnosis |  |  | 8520-3,8525 | 19110, 19120, 19125, 19160, 19162 |  |
| Mastectomy | In the 9 months after diagnosis |  |  | 8541-8 | 19180, 19182, 19200, 19220, 19240 |  |
| Axillary LN dissection | 3 mos before to 1 year after |  |  | 4023, 4051, 8543, 8547 | 38740, 38745, 19162, 19200, 19220, 19240 |  |
| Chemotherapy (any agent) | In the 9 months after diagnosis |  | V581, V662, V672 | 9925 | 96400-96549, J9000-9, Q0083-5 | 0331, 0332, 0335 |
| Doxorubicin |  |  |  |  | J9000-1, J9180 |  |
| Paclitaxel |  |  |  |  | J9170, J9265 |  |
| **Preventive care and interaction with healthcare system** | |  |  |  |  |  |
| No. physician visits | In the year prior to diagnosis | All |  |  |  |  |
| Screening mammography | In the year prior to diagnosis |  | V7611, V7612 | 8737, 8736 | 77055-6, 77058-9, 76090-2, G0202, G0204, G0206 | 0401, 0403 |
| Influenza vaccine | In the year prior to diagnosis |  | V0481 |  | 90658, G0008 |  |
| **General health status** |  |  |  |  |  |  |
| Any hospital admission | In the year after diagnosis | Inpatient |  |  |  |  |
| Charlson comorbidity score | In the year prior to diagnosis up to 1 month after diagnosis | Per prior studies^a^ |  |  |  |  |
| *Abbreviations: CPT Common Procedural Terminology; ICD-9 International Classification of Diseases, Ninth Revision; LN lymph node; mos months; No. number.* | | | | | | |
| ^a^ Klabunde CN, Potosky AL, Legler JM, Warren JL. Development of a comorbidity index using physician claims data. J Clin Epidemiol 2000;53:1258-1267. | | | |  |  |  |

**Table S2. Prediction Equations. Parameter estimates for stage IV versus stages I-III disease and for stage I/II versus stage III disease.**

| **Model to Predict Stage IV Disease** | | |  | | **Model to Predict Stage I/II Disease** | |  |
| --- | --- | --- | --- | --- | --- | --- | --- |
| **Predictor Variable** | **Parameter Estimate** | |  | | **Predictor Variable** | **Parameter Estimate^b^** |  |
| Intercept | -4.49 | |  | | Intercept | 5.90 |  |
| Metastatic disease | 2.54 | |  | | Axillary LN involvement | -2.15 |  |
| Axillary LN involvement | 0.58 | |  | | Axillary LN dissection | 0.64 |  |
| Axillary LN dissection | -1.77 | |  | | Black race | -0.37 |  |
| Age^a^ | 0.02 | |  | | Age | -0.05 |  |
| No. visits to surgeon^a^ | -0.03 | |  | | No. visits to surgeon^a^ | -0.01 |  |
| No. visits to medical oncologist^a^ | 0.02 | |  | | No. visits to medical oncologist^a^ | -0.01 |  |
| No. visits to radiation oncologist^a^ | -0.01 | |  | | No. visits to radiation oncologist^a^ | -0.02 |  |
| Radiation therapy | 0.39 | |  | | Radiation therapy | -1.09 |  |
| Breast conserving surgery vs Mastectomy | -0.69 | |  | | Breast conserving surgery vs Mastectomy | 1.81 |  |
| No surgery vs Mastectomy | 0.92 | |  | | No surgery vs Mastectomy | -1.29 |  |
| Chemotherapy (any agent) | 0.79 | |  | | Chemotherapy (any agent) | -1.12 |  |
| No. physician visits^a^ | -0.02 | |  | | No. physician visits^a^ | 0.01 |  |
| Screening mammography | -0.63 | |  | | Screening mammography | 0.64 |  |
| Influenza vaccine | -0.05 | |  | | Influenza vaccine | 0.30 |  |
| No. hospital admission (for any cause)^a^ | 0.10 | |  | | No. hospital admission (for any cause)^a^ | -0.14 |  |
| Charlson score: None vs Unknown | -0.42 | |  | | Charlson score: None vs Unknown | 0.54 |  |
| Charlson score: 1 vs Unknown | -0.33 | |  | | Charlson score: 1 vs Unknown | 0.49 |  |
| Charlson score: 2 or more vs Unknown | -0.25 | |  | | Charlson score: 2 or more vs Unknown | 0.31 |  |
| Parameter estimates shown for ease of applicability. | | |  | |  |  |  |
| Model fit characteristics: R-square 0.13, Max-rescaled R-square 0.46 | | |  | | Model fit characteristics: R-square 0.21, Max-rescaled R-square 0.41 | |  |
| Model fit for single predictor: R-square 0.08, Max-rescaled R-square 0.26 | | | | Model fit for single predictor: R-square 0.12, Max-rescaled R-square 0.22 | | | |
| *Abbreviations: LN lymph node; No. number* | | |  | |  |  |  |
| ^a^ Entered as continuous variables. | |  |  | |  |  |  |
| ^b^ Parameter estimates for predicting Stage III disease = -(parameter estimate for  Stage I/II disease). | | |  | |  |  |  |

**Table S3. Test Characteristics After Applying Prediction Equations on Validation Set Samples**

|  |  | **Validation Set: Predicting Stage I/II** | | | |  | **Validation Set: Predicting Stage III** | | | |  | **Validation Set: Predicting Stage IV** | | | |
| --- | --- | --- | --- | --- | --- | --- | --- | --- | --- | --- | --- | --- | --- | --- | --- |
| **Probability Cutpoint** |  | **Sensitivity** | **Specificity** | **PPV** | **NPV** |  | **Sensitivity** | **Specificity** | **PPV** | **NPV** |  | **Sensitivity** | **Specificity** | **PPV** | **NPV** |
| **0.05** |  | 100 | <1 | 91 | - |  | 88 | 69 | 22 | 98 |  | 81 | 89 | 24 | 99 |
| **0.10** |  | 100 | <1 | 91 | 88 |  | 78 | 83 | 30 | 98 |  | 70 | 93 | 31 | 99 |
| **0.15** |  | 100 | 2 | 91 | 85 |  | 71 | 88 | 36 | 97 |  | 61 | 96 | 39 | 98 |
| **0.20** |  | 100 | 4 | 91 | 84 |  | 63 | 91 | 40 | 96 |  | 55 | 97 | 46 | 98 |
| **0.25** |  | 100 | 9 | 92 | 81 |  | 56 | 93 | 43 | 96 |  | 50 | 98 | 51 | 98 |
| **0.30** |  | 100 | 12 | 92 | 78 |  | 50 | 94 | 46 | 95 |  | 45 | 98 | 55 | 98 |
| **0.35** |  | 99 | 16 | 92 | 74 |  | 44 | 96 | 50 | 95 |  | 41 | 99 | 59 | 97 |
| **0.40** |  | 99 | 20 | 93 | 70 |  | 39 | 97 | 53 | 94 |  | 38 | 99 | 62 | 97 |
| **0.45** |  | 99 | 25 | 93 | 66 |  | 34 | 97 | 56 | 94 |  | 35 | 99 | 63 | 97 |
| **0.50** |  | 98 | 29 | 93 | 63 |  | 29 | 98 | 60 | 94 |  | 32 | 99 | 65 | 97 |
| **0.55** |  | 98 | 34 | 94 | 59 |  | 25 | 99 | 63 | 93 |  | 28 | 99 | 68 | 97 |
| **0.60** |  | 97 | 39 | 94 | 56 |  | 20 | 99 | 65 | 93 |  | 24 | 100 | 70 | 97 |
| **0.65** |  | 96 | 44 | 95 | 52 |  | 16 | 99 | 68 | 92 |  | 20 | 100 | 70 | 97 |
| **0.70** |  | 95 | 50 | 95 | 48 |  | 12 | 100 | 72 | 92 |  | 16 | 100 | 72 | 96 |
| **0.75** |  | 93 | 56 | 96 | 45 |  | 9 | 100 | 74 | 92 |  | 12 | 100 | 74 | 96 |
| **0.80** |  | 91 | 63 | 96 | 41 |  | 4 | 100 | 76 | 92 |  | 8 | 100 | 74 | 96 |
| **0.85** |  | 88 | 71 | 97 | 37 |  | 2 | 100 | 79 | 91 |  | 5 | 100 | 79 | 96 |
| **0.90** |  | 83 | 78 | 98 | 31 |  | <1 | 100 | 78 | 91 |  | 2 | 100 | 82 | 96 |
| *Abbreviations: NPV= negative predictive value, PPV= positive predictive value* | | | | |  |  |  |  |  |  |  |  |  |  |  |
